# Charting a New Era to Modernize United States Infant Formula Regulation

**DOI:** 10.1016/j.advnut.2026.100606

**Published:** 2026-02-14

**Authors:** Douglas G Burrin, Amy B Hair, Sharon M Donovan

**Affiliations:** 1 USDA/ARS Children’s Nutrition Research Center, Section of Pediatric Gastroenterology, Hepatology and Nutrition, Department of Pediatrics, Baylor College of Medicine, Houston, TX, United States; 2 Division of Neonatology, Department of Pediatrics, Baylor College of Medicine, Texas Children’s Hospital, Houston, TX, United States; 3 Department of Food Science and Human Nutrition, University of Illinois Urbana-Champaign, Urbana, IL, United States

Proper nutrition in the first year of life is critical for supporting infant growth and development. Human milk (HM) is the biological norm for infants; however, 73% of United States infants receive infant formula (IF) at some point in the first year [1]. As IF may provide sole-source nutrition during the first 4 to 6 mo, its nutritional composition and microbial safety are paramount. Prompted by the 2022 formula shortages and emerging safety concerns, the United States Food and Drug Administration (FDA) launched “Operation Stork Speed” in March 2025 to overhaul IF regulations that have become outdated and have lagged international consensus since the Infant Formula Act (IFA) was established in 1980. In June 2025, experts from academia, industry, and community stakeholders were convened to address IF nutrient specifications, regulatory frameworks, safety protocols, marketing practices, and specialized applications. Three articles in this issue of *Advances in Nutrition* [[2], [3], [4]] outline IF nutrient composition, regulatory/safety issues, and marketing/breastfeeding support and offer a comprehensive blueprint for aligning United States standards with global evidence. These insights underscore the urgent need for evidence-based updates to enhance infant nutrition and health outcomes, reduce disparities, and foster innovation while prioritizing HM as the gold standard.

## Realigning Nutrition with HM Biology

*Part 1: Nutrient Considerations* [2] describes the current FDA guidelines, highlights inadequate standards, and raises concerns about the levels of several macronutrients. For example, current FDA guidelines mandate minimum linoleic acid levels but lack maximal limits or requirements for DHA and arachidonic acid, despite international consensus for their addition based on their roles in neurocognitive and visual development. Some manufacturers voluntarily add arachidonic acid and DHA, but the lack of a mandate and specific maximums for linoleic acid can result in IF fatty acid profiles differing from those of HM.

Carbohydrate sources are also highlighted, given their role as a major energy source and a key substrate for the gut microbiota. Surprisingly, over half of United States IF substitute lactose, the primary HM carbohydrate, with glucose polymers such as corn syrup solids, which have been linked to excess weight gain and altered gut microbiota in observational studies [5]. In IF for preterm infants, corn syrup solids are included at higher levels than lactose due to concerns about osmolality and the developmental capacity for digestion. The IFA does not specify the type or level of carbohydrate; only that the ingredients must be generally recognized as safe (GRAS) for use in IF. Thus, other sugars may be used based on cost, solubility, and concerns about lactose intolerance, although it is rare in healthy term infants. The panel questions whether corn syrup solids should be considered added sugars, since the 2020–2025 Dietary Guidelines for Americans do not recommend these sugars for infants aged <2 y [6]. Given the available evidence, perhaps lactose-reduced formulas should be reserved for specific medical indications rather than the general population.

Oligosaccharides are another significant form of carbohydrate that are enriched in HM (>200 molecular forms) compared with cow’s milk. These HM oligosaccharides function as prebiotics, selectively feeding gut bacteria, such as *Bifidobacterium* species. Only a few HM oligosaccharides have been added voluntarily, as there is no requirement or guidance for inclusion in IF, other than GRAS status. Lastly, the protein content of IF exceeds that of HM and has been considered a factor driving greater weight gain and adiposity in formula-fed infants, perhaps due to branched-chain amino acid-induced insulin-like growth factor I production [7] as shown in clinical trials of IF with reduced protein content [8]. Thus, considering the IF protein limits established by the European Society for Pediatric Gastroenterology Hepatology, and Nutrition, together with a priority on lactose, may better align with the physiological growth patterns of breastfed infants.

## Modernizing the Safety and Regulatory Process

*Part 2: Regulatory and Safety Considerations* [3] reviews the regulatory framework for new ingredients entering the United States IF market via the GRAS review process, in which safety is of primary concern. The panel raised issues that limit the entry of new formulas, such as cost, strict growth monitoring studies, protein quality standards, and market restrictions due to the Women, Infants, and Children program, which may have contributed to the 2022 formula shortage. This prompted a National Academies of Sciences, Engineering, and Medicine review that recommended a public formula registry to enhance oversight. Additionally, although Europe enforces strict maximum residue limits for contaminants in IF, the United States lacks enforceable federal thresholds and relies instead on voluntary monitoring under the “Closer to Zero” initiative. Contaminant risks pose amplified threats to infants due to immature detoxification systems. For these safety reasons, as well as providing a trusted source of formula information, the panel recommended a centralized, FDA-maintained database of IFs to support caregivers and health care professionals in making evidence-based decisions. To address these safety gaps, experts advocate replacing outdated testing that focuses narrowly on protein and growth with New Approach Methods. These New Approach Methods include high-throughput screening using human infant gut enteroids and neonatal swine models, which may offer more predictive alternatives for functional and toxicity assessment of new IF ingredients and finished products.

## Nutrition for Preterm Infants in the Neonatal Intensive Care Unit

Although Operation Stork Speed primarily focuses on term IF, many infants in the neonatal intensive care unit (NICU)—both term and preterm—require IF to meet their nutritional needs. Importantly, preterm IF is currently exempt from the IFA but would benefit from regulatory guidance on nutrient levels and the addition of bioactive components. The FDA expert panel considered the needs of these infants in *Part 3* of this series [4]. Many of the concerns raised for term IF are relevant in the NICU; however, the formula options are more limited. Additionally, only 2 IFs are routinely used for preterm infants after NICU discharge [9], both of which contain corn syrup solids, despite limited evidence supporting their use [2]. NICU practices prioritize feeding HM, especially mothers’ own milk (MOM); however, not all mothers can provide sufficient HM. Pasteurized donor HM is recommended as a supplement to MOM for preterm infants but is not routinely accessible in all NICUs [10]. Thus, IF remains the only feasible feeding option for these vulnerable infants when MOM and donor HM are unavailable. Building on the themes highlighted by the FDA expert panel, these observations emphasize ongoing challenges in the availability, regulation, and development of nutrition products and the critical need for continued research and development to expand the diversity of specialized preterm IF.

Collectively, these articles call for Operation Stork Speed to serve as a catalyst for modernizing an outdated system to evaluate the nutritional adequacy and safety of IF in the United States. Implementing these recommendations could promise products that are nutritionally superior and safer. For clinicians and researchers, the recommendations offer a framework for evaluating IF using more advanced preclinical and clinical approaches. As nutritional science and global standards advance, modernizing this system is not just a regulatory necessity; it is a safeguard for our most valuable resource, the healthy development of our children.

## Author contributions

The authors’ responsibilities were as follows – DGB, ABH, SMD: contributed equally to the writing and editing; and all authors: read and approved the final manuscript.

## Declaration of Generative AI and AI-assisted technologies in the writing process

During the preparation of this work the authors used GeminiPro to improve the clarity and word limit of the manuscript. After using this tool/service, the authors reviewed and edited the content as needed and take full responsibility for the content of the publication.

## Funding

This project was supported in part by federal funds from the USDA, Agricultural Research Service under cooperative agreement number 3092-51000-060-01 (DGB), NIDDK DK138032 and NICHD HD112396 (SMD), and NIDDK DK135602 (ABH).

## Conflict of interest

SMD reports a relationship with ByHeart Inc that includes board membership. SMD reports a relationship with DSM Nutritional Products Inc that includes: speaking and lecture fees. SMD reports a relationship with Abbott Nutrition that includes: consulting or advisory and speaking and lecture fees. SMD reports a relationship with Perrigo Company that includes: speaking and lecture fees. SMD reports a relationship with Nestlé SA that includes: funding grants. SMD reports a relationship with Arla Foods that includes: consulting or advisory. SMD and DGB both serve as Co-chairs of the Infant Nutrition Science Coalition (INSC) steering committee. The INSC brings together scientific experts from academia, government, and industry to advance research on human milk and infant nutrition and is coordinated by International Life Sciences Institute (ILSI) United States and Canada, in partnership with Oregon State University – SMD. If there are other authors, they declare that they have no known competing financial interests or personal relationships that could have appeared to influence the work reported in this paper. ABH has nothing to disclose.
